# Olive Pomace Inclusion Alters the Microbial Community of Black Soldier Fly Larvae Frass While Maintaining Fertilizer Quality

**DOI:** 10.1002/mbo3.70180

**Published:** 2025-11-30

**Authors:** Ivã Guidini Lopes, Nathali Machado de Lima, Teresa Ribeiro, Daniel Murta, Jean Wan Hong Yong, Cecilia Lalander

**Affiliations:** ^1^ Department of Biosystems and Technology Swedish University of Agricultural Sciences Alnarp Sweden; ^2^ Centre for Ecosystem Science, School of Biological, Earth and Environmental Sciences Sydney Australia; ^3^ Ingredient Odyssey SA ‐ Entogreen Várzea Portugal; ^4^ Egas Moniz Center for Interdisciplinary Research (CiiEM), Egas Moniz School of Health & Science Almada Portugal; ^5^ Department of Energy and Technology Swedish University of Agricultural Sciences Uppsala Sweden

**Keywords:** 16S, agriculture, ITS, microbiome, organic fertilizer

## Abstract

Olive pomace (OP) is a sludge arising from the production of olive oil, generated in increasing amounts in Portugal. The management of this toxic waste stream is complex and the number of processing plants is limited. In this study, OP was incorporated as a feed component for rearing black soldier fly larvae (BSFL) under industrial conditions. Larvae were reared inside a climate‐controlled room with regulated temperature and humidity. The rearing cycle lasted 13 days, after which larvae were harvested. In addition to assessing bioconversion efficiency and larval proximate composition, the resulting frass was examined for its fertilizer potential. Frass was analyzed for plant nutrient content and microbial profile in three forms: fresh, heat‐treated (70°C for 1 h), and pelletized. The inclusion of OP in the diets reduced waste‐to‐biomass conversion efficiency (21.5%_DM_ to approximately 13.3%_DM_) but did not affect the proximate composition of the larval biomass, which consistently contained around 43%_DM_ crude protein and 20%_DM_ crude fat. Neither the presence of OP nor the applied post‐treatments altered the nutrient composition of frass, which contained on average 3.5% total N, 2.6% P_2_O_5_, and 5.9% K_2_O. However, at the highest inclusion level (84%), the abundance of bacterial and fungal groups was significantly reduced. The predominant phyla in the frass were Actinobacteria, Bacteroidota, Firmicutes, Proteobacteria, Ascomycota and Basidiomycota, and the dynamics of microbial communities were influenced by specific micronutrients. The presence of OP led to a significant reduction of potentially pathogenic bacteria and fungi in the frass, indicating a sanitizing effect attributable to this material.

## Graphical abstract

Olive pomace was evaluated as a feed component for rearing BSF larvae in industrial scale. The presence of olive pomace did not affect larvae or frass composition, even when frass was submitted to heat treatment or pelletization. Bacterial and fungal communities were affected by olive pomace.

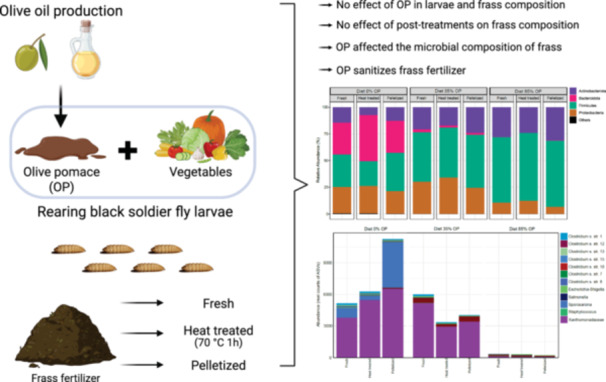

## 1 Introduction

Agricultural production in the Mediterranean biogeographic region is based on the cultivation of specific crops that are adapted to its unique climatic conditions with warm, dry summers and mild, rainy winters. Among the most cultivated crops in that region are grapes, citrus fruit, some grains and olives (*Olea europaea* L.), with both Spain and Portugal together accounting for more than 65% of the European olive oil production (European Commission 2020). As a consequence, large amounts of olive pomace—the main solid by‐product deriving from olive oil extraction, composed of olive seeds and pulp—are generated in those countries, with an average of 400 kg of olive pomace obtained for every ton of processed olives (Tapia‐Quirós et al. 2020). Although some applications to olive pomace have been explored—such as a valuable biomass for energy production (Azzaz et al. 2020) or a raw material for feed production (European Commission 2017)—the large amounts generated annually, combined with the high pollution potential (mostly due to the presence of phenolic compounds, pesticides, mycotoxins and polycyclic aromatic hydrocarbons) (Schmidt et al. 2023), suggests that alternative usages should be considered.

Olive pomace has been treated by several established methods, including thermophilic composting, thermal treatments, anaerobic digestion and others (Muscolo et al. 2019). More recently, it was proposed that a bioconversion treatment using black soldier fly (*Hermetia illucens*, BSF) larvae could also be used to treat olive pomace, especially if it was mixed with other materials of organic origin. Ramzy et al. (2022) studied the bioconversion of olive pomace mixed with wheat bran at distinct inclusion levels (0, 25, 50% and 75%) and demonstrated that it can be considered a feasible feed component for the larvae, with minimum negative impact to the larvae' development. However, at a high inclusion level (75%) of pomace, the authors reported lower material reduction, larval growth and protein accumulation, but at lower levels (25%–50%), the proximate composition of the larval biomass remained unaffected. Similar findings were reported by Ameixa et al. (2023), who observed reduced substrate conversion and lower protein content in the larval biomass. Those authors also documented a significant shift in the larvae's fatty acids profile: lauric acid (C12:0), a medium‐chain saturated fatty acid markedly decreased, while oleic acid (C18:1) accumulated as higher proportions of olive pomace were included in the diet. Both studies further reported prolonged development times, with pupation being delayed at olive pomace inclusion levels above 50%.

The bioconversion of biowaste with BSF larvae generates not only a larval biomass that can be used as an animal feed ingredient (Osuch et al. 2024) but also a treatment residue (called frass), which can be used as an organic fertilizer (Lopes et al. 2022). According to the most recent definition by the European Union, frass is the common designation used to describe “insect larvae feces”, also including the feeding substrate, parts of farmed insects and dead eggs (European Commission 2021). In addition, frass was recently placed within the EU Regulation 142/2011 as an animal manure, requiring a heat treatment of 70°C during 1 h before commercialization, to ensure biological safety (European Commission 2011). Similarly to what is normally observed in the larval biomass, the composition of the frass is also affected by the type of feed substrate provided to the larvae, in relation to plant nutrients, bioactive compounds and microbial composition (Gold et al. 2020; Lopes et al. 2022). Even though the bioconversion of olive pomace with BSF larvae have been investigated before (in small scale experiments), the effects of the dietary inclusions of pomace in the resulting frass remain unknown, both in terms of how the nutrient composition and the microbiome of frass might change when including this biowaste in larval feed.

Frass has been shown to have wide‐ranging positive effects on soil‐plant systems, which are believed to be associated with its high concentrations of organic carbon, nitrogen and phosphorus (Beesigamukama et al. 2022) and its varied microbial composition that is known to stimulate microbial activity in soils (Esteves et al. 2022). Studies have also indicated that the presence of chitin in frass (from larvae molts) can exert positive effects in plants, including protection against pests and improved plant growth, a process that seems to be mediated by microorganisms (Barragán‐Fonseca et al. 2023). The benefits of fertilizers such as frass to the soil‐plant system are strongly dependent on several factors: for example the origin of the material, its nutrient and microbial composition, the potential presence of phytotoxic compounds and the application dose (Abbott et al. 2018; Chen et al. 2018).

In a recent study, Praeg and Klammsteiner (2024) demonstrated that frass from BSF larvae, yellow mealworms (*Tenebrio molitor*) and Jamaican field cricket (*Gryllus assimilis*) stimulated soil microbial activity, asserted by increased microbial respiration and microbial biomass carbon. This was observed in both fresh and heat‐treated frass, which was partially unexpected, seen that a 70°C heat treatment for 1 h can inactivate selected microbial groups, for example pathogenic microorganisms such as *Salmonella* spp. and *Clostridium* spp. (Van Looveren et al. 2022). It would thus be feasible to assume that other, more beneficial microbial groups, could be negatively affected as well. Interestingly, those authors verified that even after the heat treatment, frass still boosted the soil's microbial activity, indicating that this fertilizer's contribution to soil microbiota and consequently plant growth might be strongly linked to its nutrient content (Praeg and Klammsteiner 2024).

To the authors knowledge, the inclusion of olive pomace in BSF larvae diets was investigated in relation to its effects on the bioconversion process performance (using small scale settings) and proximate composition of reared larvae, while the pomace‐derived frass composition remains a knowledge gap. In this study, the goal was to evaluate the effects of olive pomace, included as part of the larvae's diet in different inclusion levels, on process performance, larval biomass quality and composition (both in terms of nutrients and microorganisms) of the resulting frass fertilizers. Complementarily, the evaluated frass was analyzed both fresh (without any post‐treatment) but also after being submitted to two different post‐treatments methods—heat‐treatment at 70°C for 1 h and pelletization, to evaluate if these post‐treatments would exert any effect on the composition of this fertilizing material. It was hypothesized that olive pomace would not interfere negatively with the bioconversion process but that its presence in the larvae diets could affect the microbial composition of frass, and that the resulting frass would have similar concentration of nutrients to those reported in the literature (Gärttling and Schulz 2022; Lopes et al. 2022). The obtained results partially confirmed the hypothesis, since olive pomace affected bioconversion efficiency (BCE) differently at distinct inclusion levels, as well as the final microbial composition of frass.

## 2 Material and Methods

### 2.1 Larvae and by‐Products

Newly hatched black soldier fly (BSF) larvae were reared for approximately 5 d in Gainesville diet (30% dry matter, DM), at an industrial BSF rearing unit located in Santarém, Portugal. These young larvae (4.6 ± 2.1 mg) were counted and separated into batches according to the company's procedures, for further use in the study. Briefly, the procedure for young larvae separation and use was taken as follows: larvae originating from approximately 12.6 g of eggs were reared in a box containing Gainesville diet and, after hatching and rearing them for 5 d, the box's content was divided into 18 portions of larvae (mixed with the consumed diet), with one portion being used in one replicate (plastic boxes of 40 × 60 x 12 cm) during the bioconversion process (see details of the experimental design in Section 2.2). The by‐products used as feed substrates for the larvae in this study were vegetable cuttings from a local processing plant (red peppers, pumpkin, broccoli and zucchini) and olive pomace from a local olive oil processing industry. Both sites were located within 10 km of the experimental site where the experiment took place. These by‐products are normally delivered weekly to the company and were provided fresh, without prior freezing or processing. All by‐products were ground in an industrial grinder to reduce particle size to a maximum of 3 mm and blended before being provided to the larvae.

### 2.2 Experimental Design

**Figure 1:**
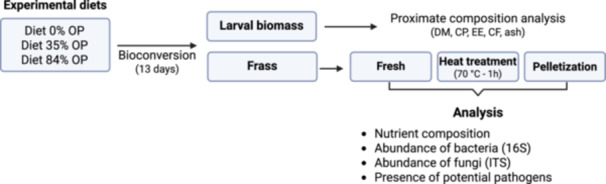
Diagram summarizing the experimental design adopted in this study. The experimental diets were bioconverted by black soldier fly larvae for 13 days, followed by separation of larval biomass and frass by mechanical sieving. While the larval biomass was promptly analyzed for its proximate composition, frass was sampled either fresh, heat treated (70°C for 1 h) or pelletized, followed by nutrient composition analysis and microbiome profiling.

Three experimental diets were assembled in this study using vegetable cuttings and a grain mix (Gainesville diet) as the base ingredients: the control diet contained only the vegetables and grains (Diet 0% OP, which stands for “olive pomace”); the second diet had an inclusion level (wet basis) of 35% of olive pomace (Diet 35% OP) and the third diet comprised 84% olive pomace (Diet 84% OP) (Figure 1). The diets were formulated to have a proximate composition that would fall within expected ranges of nutrients needed to rear BSF larvae, according to the company's protocols. The crude protein of diets ranged between 5.6% and 7.1%, crude fat between 1.8% and 3.5% and crude fiber between 9.6% and 13.6%. The dry matter (DM) content of the diets was 29.9 ± 3.2%. Details about diets' composition and their nutritional values are presented in the Supplementary Material (Table S1). For each experimental diet, 18 replicates (plastic boxes) were assembled.

#### 2.2.1 BSF Larvae Bioconversion Step

The bioconversion process was performed in the aforementioned plastic boxes, which had a useful area of 2400 cm^2^ each. A total of 13.5 kg (wet weight) of each diet was placed inside each replicate (reaching an average depth of 6.1 ± 0.7 cm), together with individual portions of larvae, prepared as described in Section 2.1. The experiment was conducted inside a climatic chamber with controlled temperature (25 ± 1°C) and humidity (70 ± 10%), located at the Entogreen's research center in Santarém, Portugal, which simulates exactly what is carried out in an industrial plant for processing by‐products with BSF larvae. The bioconversion process for all diets lasted for 13 days, upon which the larvae and frass were separated by sieving, using a circular vibrating mechanical sieve (2–10 mm mesh). The weight of larvae and frass collected from each replicate was recorded, as well as the weight of 10 randomly selected larvae, which were used to estimate the individual larval weight and number of larvae remaining at the end of treatment.

#### 2.2.2 Post‐treatment of Frass

The frass from six randomly selected replicates per treatment was divided into three portions. One “*fresh frass*” sample (frass collected immediately after sieving) was collected directly after harvest, while two other samples were subjected to different post‐processing (Figure 1) techniques: a *heat treatment* following the proposed standards of the EU Regulation 142/2011 (European Commission 2011) (70°C for 1 h) and a mechanical pelletization process, to assess the impact of the post‐treatments on the composition and microbial community of the samples. For the heat treatment, an oven was set to 70°C and the frass sample was placed on top of sterile paper in thin layers (1 cm) for 1 h, while for the pelletization, a pellet machine (Power Classic 380 W) was used. Before its use and in between batches of frass (replicates of all treatments), the machine was surface sterilized by spraying ethanol 70% and allowing it to dry naturally. Frass was added to the machine slowly and continuously, to maintain a flow of homogeneous pellets being produced (Figure S1). The pellets were left still on top of a plastic tarp for approximately 1 h for cooling down and then sampled for further analysis.

### 2.3 Sample Collection and Analysis

The initial feed substrates were collected (one single sample) after the grinding and mixing processes described in Section 2.2 and analyzed initially for DM and volatile solids (VS) content. This was done by drying the samples in an oven (65°C for 48 h) and further burning them in a muffle oven as suggested by Guidini Lopes et al. (2023). Samples were also analyzed for crude protein (using the Kjeldahl method, using sulfuric acid to convert nitrogen into ammonium, then distilled with 10 N NaOH and titrated with HCl) and using a protein conversion factor of 6.25. Crude fat was evaluated by means of the Soxhlet extraction method, where the dried sample was placed in a filter paper and continuously washed with hexane for fat dissolution (AOAC 1920), while crude fiber was analyzed by the AOAC 962.09 method, in which the fat‑free sample is sequentially boiled in dilute sulfuric acid and sodium hydroxide, followed by filtration, washing, drying, and incineration of the residue to determine fiber content (AOAC 1962). The same six randomly selected replicates mentioned in Section 2.2.2 were used for larvae and frass sample collection. Three larvae samples (300 g) were collected from each treatment. These were used for proximate composition determination (crude protein, fat, fiber and ash), analyzed in the same laboratory using the same standard methods as described above. Briefly, the larvae were analyzed for crude protein based on Kjeldahl nitrogen, but using a conversion factor of 4.76 as suggested by Janssen et al. (2017). Crude fat (ether extract) was done by the Soxhlet extraction method (AOAC 1920) and crude fiber by the same AOAC 962.09 method (AOAC 1962).

Regarding frass nutrient composition, three samples (300 g) of each frass type (fresh, heat‐treated and pelletized) were collected from each treatment and analyzed. Frass samples were analyzed for DM and VS content (as described above), total organic carbon (TOC), organic matter (OM) and concentration of plant nutrients, including nitrogen (N), phosphorus (P_2_O_5_), potassium (K_2_O), total organic carbon (TOC), C/N ratio, calcium (CaO), magnesium (MgO), copper (Cu), manganese (Mn), boron (B), zinc (Zn), sodium (NaO), pH and electrical conductivity (EC). Frass TOC and N were analyzed by combustion in an elemental analyzer (LECO CN 628). For the analysis of macro and micronutrients, samples were burned at 500°C for 4 h in a muffle oven and the remaining ashes were dissolved in 0.1 M HNO_3_ solution. Then, P_2_O_5_ was determined by spectrophotometry in the visible region, while NaO and K_2_O by flame‐photometry (using a Micronal B462 photometer). Other nutrients (Ca, Mg, Cu, Mn and Zn) were analyzed with the aid of an atomic absorption spectrophotometer (SpectrAA 220, Varian). Boron was determined by extracting the sample with hot water, reacting the extract with azomethine‐H reagent (to form a yellow complex) and then measuring the absorbance at 420 nm. Frass pH and EC were determined by diluting the sample in deionized water (using a 1:2.5 dilution) and measuring both parameters after a 60 min resting period.

Additionally, frass was collected from the six selected replicates for microbiome profiling, described in section 2.4. For microbial analysis, the pelletized frass samples were ground in a sterilized coffee grinder and sieved (2 mm mesh) to a similar particle size as the other frass samples (fresh and heat‐treated frass). Therefore, for larval proximate composition, frass nutrient characterization and frass microbiome profiling, a total of 9, 27 and 54 samples were analyzed, respectively. The samples used for microbial profiling were collected in 50 mL Falcon tubes and stored at −20°C pending analysis.

The presence of pathogenic bacteria mentioned in the European Regulation 142/2011 for frass commercialization standards (European Commission 2011) was also evaluated in the same frass samples that were analyzed physico‐chemically. *Salmonella* spp. was analyzed in 25 g of frass, estimated according to the methodology proposed in ISO 6579 (ISO 2002), and presumptive *Escherichia coli* was analyzed by means of a colony‐count technique after incubation of plates at 44°C, according to ISO 11866‐2 (ISO 2005). All analyses of frass and larvae (except the microbial profile of frass) were performed by an accredited laboratory (Mérieux NutriSciences, Vila Nova de Gaia, Portugal).

### 2.4 Microbiome Composition Analysis

The relative abundance of bacterial and fungal groups within fresh, heat‐treated and pelletized frass was investigated. To characterize the microbial community composition and diversity, samples (six samples per treatment and per type of post‐treatment) were processed at InnovPlantProtect (InPP) (Elvas, Portugal) for DNA extraction and sequencing, according to the protocols described in the Earth Microbiome Project (https://earthmicrobiome.org/protocols-and-standards/).

#### 2.4.1 DNA Extraction

The samples' DNA was extracted from 250 mg of frass according to the protocol of DNeasy of the PowerSoil Kit (Qiagen, Venlo, Netherlands), following the manufacturer's instructions. The extracted DNA was quantified and assessed for purity before sequencing.

#### 2.4.2 PCR Amplification and Sequencing

The extracted DNA underwent two separate sequencing runs. The V4 hypervariable region of the 16S rRNA gene was amplified using primers 515 F and 806 R (Caporaso et al. 2023) modified with Illumina adapter overhangs. The amplified products were purified and subjected to a second PCR for indexing, where unique Illumina Nextera XT indices were added to each sample. Paired‐end sequencing was carried out on the Illumina MiSeq platform using the MiSeq Reagents Kit v3 with a 2 × 150 bp Nano configuration. A similar workflow was used for sequencing the ITS1 hypervariable region, employing ITS1F and ITS2 primers, followed by paired‐end sequencing on the same platform with 2 × 250 bp reads.

#### 2.4.3 Bioinformatics Analysis

Raw sequence data processing was done using dada2, for 16S rRNA raw reads, the sequences were trimmed by length and quality (absence of N, maximum error rates maxEE were 2 for both forward and reverse reads). An amplicon sequence variant (ASV) was determined according to the dada2 algorithm, and chimera ASVs were removed by the “consensus” method. Taxonomic annotation was performed by the naive Bayesian classifier (provided in dada2 package, default settings) with the SILVA 138 database (Quast et al. 2013) as the training set. For ITS1 raw reads, the primer sequences were identified and removed by cutadapt (Martin 2011). Sequences were trimmed by length and quality (absence of N, maximum error rates maxEE were 2 for both forward and reverse reads). ASVs were determined according to the dada2 algorithm (version for ITS); chimera ASVs were removed by the “consensus” method. Taxonomic annotation was performed by the naive Bayesian classifier with the UNITE ITS database (Nilsson et al. 2019).

### 2.5 Process Efficiency

Process efficiency was evaluated by means of the larval yield (wet basis), which is the biomass of larvae produced in each replicate, and by two standard indexes, namely the bioconversion efficiency (BCE, Eq. 1), which gives the amount of waste substrate converted into larval biomass; and the material reduction (MatRed, Eq. 2), which gives the reduction of the feed substrate provided to the larvae (i.e. is converted into frass). Both indexes were calculated according to the equations described in Guidini Lopes et al. (2023) as present below:

(1) $$\text{BCE}=\left(\frac{{\text{m}}_{\text{lv}}}{{\text{m}}_{\text{initial}}}\right)\times 100$$

where *m*_lv_ and *m*_initial_ are the total mass of harvested larvae and the initial substrate, respectively.

(2) $$\text{MatRed}\;=\;\left(1\;-\;\frac{{\text{m}}_{\text{frass}}}{{\text{m}}_{\text{initial}}}\right)\times 100$$

where *m*_frass_ and *m*_initial_ are the total mass of frass and the initial substrate respectively.

### 2.6 Statistical Analysis

Process parameters indexes (larval yield per experimental unit, BCE and MatRed) and larvae proximate composition (DM, ash, crude protein, fiber and fat) data were first analyzed using Shapiro‐Wilk's and Levene's tests to verify the assumptions of normality and homoscedasticity, respectively. The data was found to be homogeneous and normally distributed, thus a *one‐way* analysis of variance (ANOVA) was carried out, followed by the Tukey´s *post‐hoc* multiple comparisons test with a 5% significance level. The frass composition of plant nutrients was also assessed for normality and homoscedasticity. In case the assumptions of normality and homoscedasticity were met, a factorial ANOVA followed by the Tukey's *post‐hoc* test was performed to identify differences in the frass composition within two factors: diet type and posttreatment methodology.

Statistical analyses of the microbiome data were performed using R (v4.3.1; Core 2023). Boxplots containing the relative abundance of the microbial community, and the soil chemical components proportions were plotted using “ggplot2” (v3.4.3) (Wilkinson 2011). The “phyloseq” package (v1.32.0, (McMurdie and Holmes, 2013) was used to subsample the number of reads per sample and create an object that was used for downstream analysis. Shannon and Inverse Simpson diversity indexes were calculated using the function “alpha” available through the package “microbiome” (v1.10.0) (Lahti and Shetty 2012‐2019). Differences between treatments were analyzed within a linear model framework, followed by the “emtrends” function. Differences in beta diversity were evaluated using the function “adonis” and “pairwise.adonis” from the “vegan” package (v2.5‐7, Oksanen et al. 2020) on the Bray‐Curtis dissimilarity matrix. The same matrix was used to generate a nonmetric multidimensional scaling (NMDS) analysis with the vegan function “metaMDS” and show the distribution of the community composition. Redundancy analysis (RDA) was performed to explore the relationships between microbial community composition and environmental variables. Variables were preselected using the “bioenv” function from the “vegan” package, which identifies the subset of environmental variables most strongly associated with community composition. Additionally, the “ordistep” function was applied to refine the selection. To assign functionality, the identified genera were cross‐referenced with the FungalTraits database (Põlme et al. 2020), and their primary lifestyle was determined for presentation. Additionally, specific bacterial taxa known to pose risks to soil, animal, plant, or human health were identified from the 16S data, and their relative abundances were examined.

## 3 Results

### 3.1 Process Efficiency and Larval Composition

The larval yield per treatment unit and the BCE were highest in the control with no olive pomace (OP) (2.0 kg larvae box^‐^¹ and 22%_DM_, respectively) and lowest in the diet with 35% olive pomace (Diet 35% OP) (1.6 kg box^‐1^ and 12%_DM_, respectively) (Table 1). Material reduction (MatRed) varied among treatments, with the lowest reduction observed in the 84% olive pomace diet (38%_DM_). In contrast, diets with lower olive pomace content showed higher material reductions: 55% DM in the 0% olive pomace diet and 64% DM in the 35% olive pomace diet.

**Table 1:** Process parameters related to the BSF larvae bioconversion efficiency of three diets containing no (Diet 0% OP), low (Diet 35% OP) or high (Diet 84% OP) inclusion of olive pomace.

	BCE_DM_ (%)	MatRed_DM_ (%)	Larval Yield (kg box^‐1^)
Diet 0% OP	21.5 ± 5.0^a^	54.8 ± 8.0^a^	2.0 ± 0.2^a^
Diet 35% OP	12.0 ± 0.1^b^	64.3 ± 2.0^a^	1.6 ± 0.1^b^
Diet 84% OP	13.3 ± 1.3^b^	38.4 ± 5.6^b^	1.8 ± 0.1^b^

*Note:* Values are presented as mean ± standard deviation (*n* = 18). Different superscript letters denote significant differences (Tukey′s test, *p* < 0.05).

Abbreviations: BCE, bioconversion efficiency; DM, dry matter; MatRed, material reduction.

There was no statistically significant difference (*p* > 0.05) between the proximate composition of the larval biomass in any of the parameters evaluated, except for the DM content, which was slightly higher for the larvae reared in the control diet (Diet 0% OP), which did not contain olive pomace (36%) (Table 2). Crude protein was approximately 43%_DM_ in the larvae reared the control diet with no olive pomace and 48%_DM_ in the larvae fed Diet 35% OP (*p* > 0.05). The opposite trend was true for crude fat (EE), being lowest in larvae reared on Diet 35% OP (20%_DM_) and highest in larvae reared on the control diet.

**Table 2:** Proximate composition (on a dry matter basis) of black soldier fly larvae fed different diets containing no (Diet 0% OP), low (Diet 35% OP) or high (Diet 84% OP) inclusion of olive pomace.

	DM (%)	CP (%_DM_)	EE (%_DM_)	Ash (%_DM_)	CF (%_DM_)
Diet 0% OP	36.0 ± 2.3^a^	43.2 ± 3.8	23.6 ± 2.0	9.4 ± 0.6	9.7 ± 1.7
Diet 35% OP	30.0 ± 1.1^b^	48.4 ± 1.5	20.1 ± 0.9	10.5 ± 0.4	13.4 ± 1.5
Diet 84% OP	33.4 ± 0.8^ab^	46.6 ± 1.3	22.6 ± 0.4	9.1 ± 0.3	14.1 ± 1.3

*Note:* Values are presented as mean ± standard deviation (*n* = 3). Different superscript letters denote significant differences (Tukey′s test, *p* < 0.05).

Abbreviations: CF, crude fiber; CP, crude protein; DM, dry matter; EE,ether extract (crude fat).

### 3.2 Frass Composition and the Effects of Post‐Treatments

The DM of frass immediately after the bioconversion process was 76% in the control treatment (Diet 0% OP) and in the diet with the highest inclusion (Diet 84% OP), while it was 50% for Diet 35% (Table S3). The physico‐chemical characteristics and concentration of selected macronutrients in frass considering the factorial experimental design of the study is presented in Table 3. The adoption of either heat‐treatment or pelletization in frass samples resulted in a reduction of its moisture content, while the organic matter concentration was unaffected by both post‐processing methodologies. The control with no olive pomace (Diet 0% OP) exhibited the highest total organic carbon (TOC) content (42.6%_DM_), like that of Diet 35% OP (40.7%_DM_), and higher than Diet 84% OP (39.5%_DM_), but no significant effect of post‐treatments was observed for this parameter. The same trend was observed for P_2_O_5_, with the highest concentration being found in Diet 84% OP (3.54%_DM_) and no observed impact of post‐treatments. No effects of either diet type or post‐treatments were observed for the other parameters, including total nitrogen (N_T_), K_2_O, C/N ratio and pH (Table 3). When it comes to the concentration of other macronutrients (Ca and Mg) and micronutrients (Cu, Mn, Zn and Na), the type of diet was found to influence these parameters, while post‐treatments did not, as shown in the Supplementary Material (Table S2). The mean values obtained for each of the frass' characteristics in each experimental diet and subsequent post‐treatments are presented in the Supplementary Material (Tables S3 and S4).

**Table 3:** Factorial analysis of the physico‐chemical properties of black soldier fly larvae frass, either fresh or subjected to two post‐treatments (heat treatment and pelletization) and derived from the bioconversion of three diets containing 0% (Diet 0% OP), 35% (Diet 35% OP), or 84% (Diet 84% OP) olive pomace. Values shown for each factor level (diet and type) represent marginal means ± standard deviation, averaged over the levels of the other factor. Statistical results for main effects and interactions are based on a two‐way ANOVA.

	DM (%)	OM (%_DM_)	TOC (%_DM_)	N_T_ (%_DM_)	P_2_O_5_ (%_DM_)	K_2_O (%_DM_)	C/N ratio	pH	EC (dS cm^‐1^)
Diet									
0% OP	68.9^a^	83.6	42.6^a^	3.60	1.86^b^	5.96	11.83	8.9	10.30
35% OP	59.6^b^	82.9	40.7^ab^	3.52	2.49^ab^	5.81	11.57	8.7	10.18
84% OP	61.7^b^	83.0	39.5^b^	3.39	3.54^a^	5.98	11.70	8.9	10.38
Type									
Fresh	55.36^b^	83.4	40.9	3.44	2.68	5.97	11.92	8.9	10.12
Heat	66.55^a^	83.4	40.5	3.38	2.73	5.64	11.97	9.0	10.51
Pellet	58.4^b^	82.7	41.4	3.69	2.47	6.14	11.20	8.7	10.23
Statistics									
Diet	79.7***	8.3^ns^	246.9**	5.9^ns^	34.1***	2.2^ns^	0.9^ns^	1.5^ns^	0.6^ns^
Type	84.6***	9.5^ns^	21.7^ns^	15.1^ns^	9.3^ns^	15.4^ns^	1.7^ns^	5.5^ns^	3.8^ns^
Interaction	24.1*	3.1^ns^	21.1^ns^	3.9^ns^	26.8*	5.2^ns^	2.5^ns^	2.9^ns^	2.6^ns^

*Note:* Values shown for each factor level (diet and type) represent marginal means ± standard deviation, averaged over the levels of the other factor. Statistical results for main effects and interactions are based on a two‐way ANOVA. Different superscript letters denote significant differences (Tukey′s test, *p* < 0.05). Significance levels:

Abbreviations: DM, dry matter; K2O, potassium oxide; NT, total nitrogen; OM, organic matter; P_2_O_5_, phosphate; TOC, total organic carbon.

* *p* < 0.05;

** *p* < 0.01;

*** *p* < 0.001; ns: not significant.

### 3.3 Effects of Diets and Post‐treatment Technologies on the Microbial Composition of Frass

#### 3.3.1 Effects on the Bacterial Community

**Figure 2:**
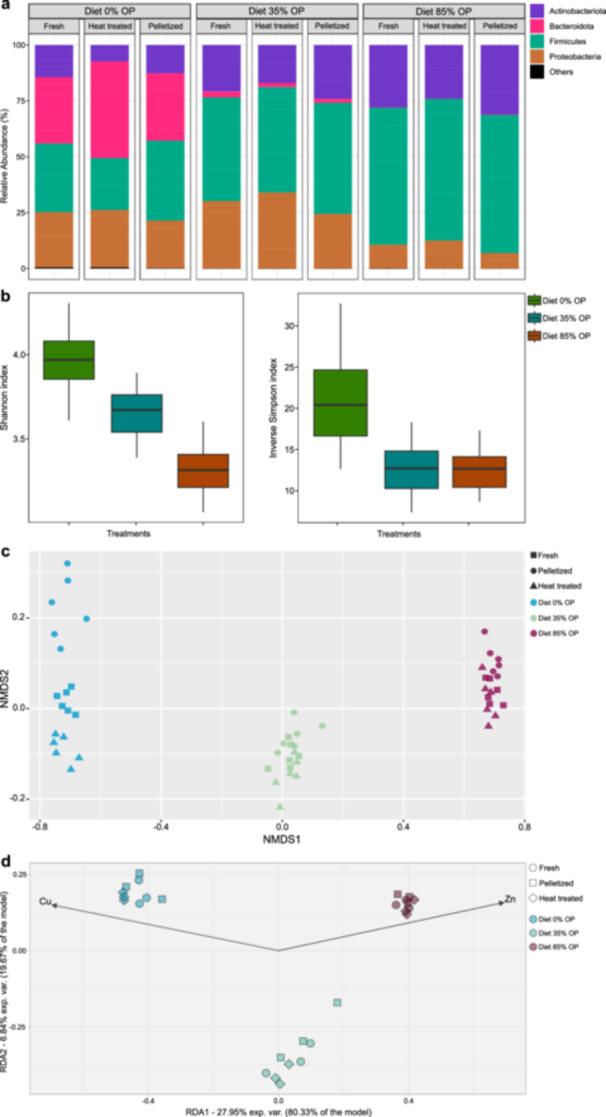
Relative abundance of bacterial groups (phylum level) (a); Shannon diversity and Inverse Simpson indexes (b); RDA displaying the correlation of bacterial groups with physico‐chemical traits of frass (c); and NMDS of bacterial groups (d) in frass types originating from the bioconversion of olive pomace (OP) with black soldier fly larvae (N = 6). The diets had no (Diet 0% OP), low (Diet 35% OP) or high (Diet 84% OP) inclusion of olive pomace.

The analysis of 16S rRNA gene amplicons resulted in a total of 1085 amplicon sequence variants (ASVs) after quality control, filtering and applying the 97% threshold. This data was used to generate the relative abundance of each identified phylum (Figure 2a). The samples contained between 16,436 and 86,900 reads and were subsampled to 16,436 reads per sample. This subsampling retained 954 ASVs, which were used for all diversity analyses. A second run targeting the ITS region and considering a clustering cut‐off of 97%, produced 553 ASVs. Total reads per sample ranged from 31,085 to 86,259 and were subsampled to 31,085 reads per sample, yielding 551 ASVs for use in all diversity analyses.

The diets significantly affected the relative abundance of bacterial groups (at phylum level) in frass, while no differences were observed in the frass samples that received distinct post‐treatments. In the control treatment (Diet 0% OP), a balanced composition of Firmicutes, Proteobacteria and Actinobacteria was observed (Figure 2a). In Diet 35% OP, a higher abundance of Proteobacteria was observed, with a very low presence of Bacteroidota. In the samples from Diet 84% OP, Proteobacteria was found in low abundance, followed by Actinobacteria, with Firmicutes being the predominant group. Irrespective of the type of post‐treatment adopted in the frass samples, the bacterial community's diversity was significantly higher in Diet 0% OP, in comparison to the other diets, according to the Shannon and Inverse Simpson diversity indexes (*p* < 0.0001). Additionally, the two diets containing olive pomace (Diet 35% OP and Diet 84% OP) were different among each other according to Shannon Diversity (*p* < 0.0001) but similar according to the Inverse Simpson index (*p* > 0.05; Figure 2b).

The PERMANOVA analysis indicated that the 16S bacterial community composition was modulated solely by the different diet types and not by the post‐treatments applied, and the finding was supported by NMDS analysis (stress: 0.0338; Figure 2c). RDA also revealed three distinct groups reflecting the different diets, with RDA1 and RDA2 collectively explaining approximately 35% of the total variance. Some of the frass' physicochemical properties, particularly copper and zinc, were identified as the only statistically significant factors by the bio.env analysis. Copper was strongly associated with Diet 1, while zinc was linked to Diet 2 (Figure 2d).

**Figure 3:**
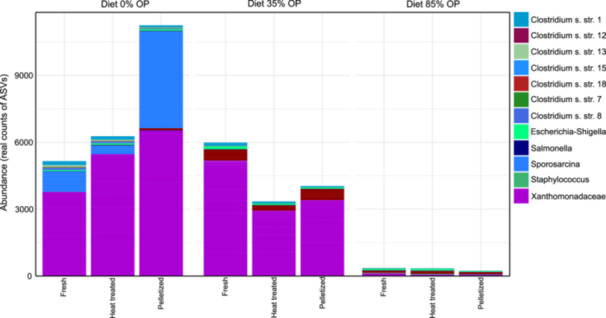
Abundance (real counts) of specific potentially pathogenic bacteria screened in fresh, heat‐treated and pelletized frass samples originated from three experimental diets containing 0%, 35% or 84% olive pomace.

The ASVs identified as belonging to taxonomic groups that can potentially be considered pathogenic, such as *Clostridium*, *Escherichia‐Shigella*, *Salmonella*, *Sporosarcina*, *Staphylococcus*, and Xanthomonadaceae, were separated from the general pool of samples, to identify potential risks of frass for the soil‐plant health continuum. Their abundance accounted for 4.41% in Diet 0% OP, 2.25% in Diet 35% OP and 0.2% in Diet 84% OP (Figure 3). The most abundantly observed microorganisms belonged to the family Xanthomodaceae, which are Gram‐negative proteobacteria considered a plant pathogen, and from the genus *Sporosarcina*, which are spore‐forming bacteria that can infect mammals. The other identified groups belonged to the clade *Clostridium*, even though it is not possible to know which genus or species those belong to.

#### 3.3.2 Effects on the Fungal Community

**Figure 4:**
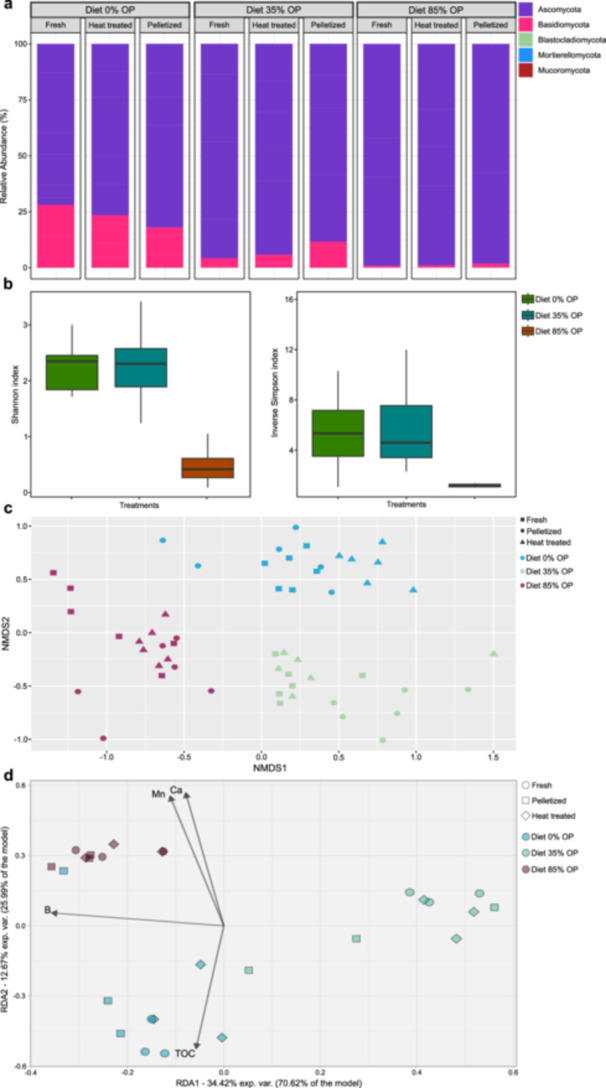
Relative abundance of fungal groups (phylum level) (a); Shannon diversity and Inverse Simpson indexes (b); RDA displaying the correlation of bacterial groups with physico‐chemical traits of frass (c); and NMDS of bacterial groups (d) in distinct frass types originating from the bioconversion of olive pomace (OP) with black soldier fly larvae (N = 6). The diets had no (Diet 0% OP), low (Diet 35% OP) or high (Diet 84% OP) inclusion of olive pomace.

In comparison to the bacterial phyla observed in the frass samples, the relative abundance of the fungal community was less diverse compared to the bacterial community (Figure 4), with an almost exclusive presence of Ascomycota in all diets, irrespective of posttreatment of frass. Low abundances of Blastocladiomycota, Mortierellomycota and Mucoromycota were also observed. There was no significant difference in fungal diversity between the control diet with no pomace and Diet 35% OP (*p* > 0.05), while such diversity was significantly higher in these diets compared to Diet 84% OP, according to both Shannon and Inverse Simpson diversity indexes (*p* ≤ 0.0001) (Figure 4b). Similarly to the findings observed for bacterial diversity, the PERMANOVA analysis indicated that the ITS fungal community composition was influenced solely by the different diet types, a finding that was also supported by the NMDS analysis (stress: 0.1686; Figure 4c). The redundancy analysis revealed a clear separation among the three diet groups, with RDA1 and RDA2 together explaining approximately 47% of the total variance. The bio.env analysis identified manganese, calcium, boron, and total organic carbon as statistically significant factors influencing the groups. Boron exhibited a negative correlation with Diet 35% OP, while calcium and manganese were strongly associated with Diet 84% OP. Total organic carbon was linked to Diet 35% OP (Figure 4d).

**Figure 5:**
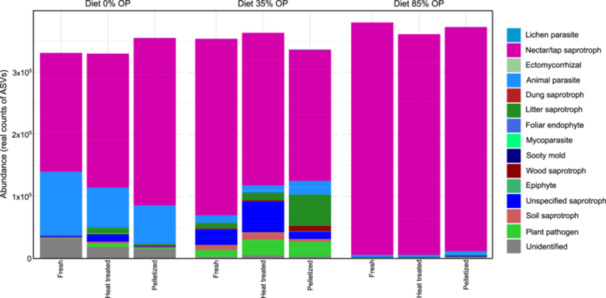
Abundance (real counts) and functionality of specific clusters of fungi, including potentially pathogenic groups, in fresh, heat‐treated and pelletized frass samples originated from three experimental diets containing 0%, 35% or 84% olive pomace.

The functionality of the fungal community was assigned using the FungalTraits database. All diets exhibited a predominance of genera recognized as nectar/tap saprotrophic in relation to other functionalities, in particular for the diet with highest inclusion of olive pomace (Diet 84% OP). In contrast, the groups found in Diet 0% OP and Diet 35% OP were majorly associated with animal parasitism and plant pathogenicity. The control diet with no olive pomace contained 23% of these groups, while 10% were found in Diet 35% OP, and 1.3% in the diet with highest inclusion of olive pomace (Diet 84% OP), thus following the same trend as observed for the bacterial groups (Figure 5).

## 4 Discussion

### 4.1 Olive Pomace Reduced Bioconversion Efficiency

The frass obtained from the bioconversion process of diets containing olive pomace (OP) visually appeared to have a higher moisture content as compared to the control with no pomace, by the time of harvest. However, upon analyses, the dry matter (DM) content of frass was only lower in Diet 35% OP (50%) (Table 3). All experimental replicates were stacked in pallets and placed in the same positions inside the three‐floors climatic chamber described in Section 2.2.1; thus, the applied ventilation inside the chamber was likely very similar in all treatments. Therefore, the differences observed in the frass' DM content can be attributed to the bioconversion process itself, i.e. to the interaction of larvae with the diets. Ameixa et al. (2023) prepared diets containing 0%–75% of olive pomace mixed with chicken feed and used it as feed substrate for BSF larvae. Those authors' results showed that the presence of olive pomace resulted in lower bioconversion efficiency (BCE) and substrate reduction (MatRed), especially at high inclusion levels ( > 50%), reaching values as low as 5.0 ± 0.1% of BCE, on a wet basis. Similarly, Ramzy et al. (2022) reported a linear decrease in MatRed (with the lowest value being approximately 17% on a wet basis), when increasing the inclusion rate of olive pomace from 0% to 75% in a wheat bran‐based diet. Both studies were conducted on a small scale, using 100–500 larvae per replicate, differing from the present study, which was conducted on an industry scale. Nonetheless, the findings of this study indicate a potential negative influence of olive pomace on the larvae's performance during bioconversion, which should be investigated in future studies.

It is likely that the olive pomace‐based diets contained phenolic compounds and tannins, possibly in high concentrations (especially in Diet 84% OP), which are substances that can be toxic to animals and plants (Wu et al. 2015). This fact might have compromised the ability of larvae to properly digest the diets containing pomace. Ameixa et al. (2023) and Ramzy et al. (2022) also hypothesized that due to the presence of distinct types of fibers (including cellulose and lignin), olive pomace's digestion by larvae could have been hampered in their studies. However, this was likely not the case for the present study, since the crude fiber concentration in the diets did not exceed 13.6% (Table S1). As demonstrated by Lindberg et al. (2025), materials such as plant residuals given exclusively to the larvae as their feed substrate, with fiber contents above 30%, certainly hamper bioconversion affecting its efficiency, compromising the process as a whole. The observation that both inclusion levels of pomace (35% and 84%) resulted in similar BCE and larvae yield was unforeseen, since a more linear effect would have been expected. Considering that this study was conducted in a large‐scale setting, in conditions that are typically less controlled than in a laboratory, it is likely to assume that the observed process efficiency parameters might have been due to normal variations observed daily in the industry. Thus, an additional experiment would clarify if indeed even a low inclusion level of olive pomace (as in Diet 35% OP) would reduce BCE and yield to the same extent. Additionally, such a reduction in process efficiency might be related to the higher level of microbial reduction observed in the frass deriving from Diet 84% OP in comparison to Diet 35% OP; however, unfolding this possible correlation is not simple, seen that during bioconversion, there is a close interaction between biological, physical and chemical factors affecting performance. Thus, it is recommended that future studies investigate broader inclusion levels of olive pomace provided for BSF larvae, in the attempt of identifying more adequate blends and the levels in which bioconversion might be compromised.

### 4.2 Larval Proximate Composition Was Not Affected by the Presence of Olive Pomace

The only difference observed in the larval biomass obtained from the three experimental diets was a slightly lower DM content in Diet 35% OP (30%_DM_) compared to the control diet with no OP (36%_DM_), while crude protein, fat, ash and fiber levels were found to be similar in all diets (Table 2). Regardless of the inclusion level of olive pomace in diets, their proximate composition was similar (Table S1), which can explain the relatively small variability observed in the obtained larval biomass amongst treatments. Similar results were reported by Ameixa et al. (2023), who observed small variations in the larval biomass in relation to crude protein (ranging from 37%_DM_ to 43%_DM_), while the crude fat content was similar in the presence or absence of olive pomace (ranging from 35.4%_DM_ to 38.5%_DM_ in that study). Due to the procedures of oil extraction performed during the production of olive oil, olive pomace is typically a product with low crude fat content, not exceeding 3% on a DM basis (Antónia Nunes et al. 2018; Ramzy et al. 2022). The substantial interest for olive pomace is mostly related to the presence of specific fatty acids and other functional compounds found in this by‐product (Difonzo et al. 2021), which can have their dynamic severely changed in the larval biomass, as reported by Ameixa et al. (2023).

### 4.3 Diet but Not Post‐Processing of Frass Affected Its Nutrient Composition

The presence or absence of olive pomace in the larval diets did not significantly influence the nutrient composition of the resulting frass, except for total organic carbon (TOC) and phosphate (P_2_O_5_), which were slightly lower and higher, respectively, in the diet with highest pomace inclusion (Diet 84% OP) (Table 3). The observed values for frass' organic matter content, total nitrogen (N_T_), potassium (K_2_O), C/N ratio and pH were found to be within the expected ranges, as reported by Lopes et al. (2022) and Gärttling and Schulz (2022). These nutritional parameters confirmed that frass can be used as a fertilizer for cultivation; however, as discussed by Lopes et al. (2024), additional parameters that are related to the stability and maturity of the frass‐fertilizer should be evaluated, as there have been reports of frass phytotoxicity. Olive pomace is rich in phenolic compounds, which are known to be phytotoxic and to inhibit plant growth and microbial development (Khdair and Abu‐Rumman 2020). These compounds can be present in frass and thus future studies should evaluate OP‐derived frass for phytotoxicity, including self‐heating capacity tests and microbial respiration assays. Mostafaie et al. (2025) investigated the impact of frass derived from BSFL bioconversion of olive pomace on soil, including seed germination bioassays using ryegrass and broccoli seeds, as well as ecotoxicological bioassays using the soil model invertebrate *Enchytraeus crypticus*. Those authors reported no significant adverse effects on the tested soil health indicators, with minor plant growth reductions being observed.

Regarding the post‐processing of frass, neither the heat treatment nor the pelletization process affected the nutrient content of the fertilizers, which were similar to the fresh frass from all three diets (Tables 3, S3 and S4). The only parameters that were significantly influenced by the post‐processing methods were the DM content of the frass, corroborating the results of Sarlaki et al. (2021), who observed a reduced DM in agro‐biowaste fertilizers after the pelletization and drying of fresh materials. Similarly, López‐Mosquera et al. (2008) evaluated the pelletization of poultry litter and found that the pelletized poultry litter had similar nutrient content as the fresh litter. This was also evaluated by Praeg and Klammsteiner (2024) for insect frass of three species, including frass from crickets (*Gryllus assimilis*), mealworms (*Tenebrio molitor*) and BSF larvae. Those authors observed that most fertilization‐related parameters in frass from these three insect species remained unchanged after heat‐treating frass at 70°C for 1 h. These results demonstrated that it is possible to heat‐treat or pelletize BSF larvae frass without losing its nutritional quality, supporting the current EU Regulation 2021/1925 for the commercialization of insect frass. In addition, it supports the feasibility of producing a pellet fertilizer, which can be easily used by farmers in comparison to applying fresh frass in powder‐form (López‐Mosquera et al. 2008).

### 4.4 Frass Microbial Composition Was Significantly Affected by the Presence of Olive Pomace in the Larval Diet

The predominance of Firmicutes, Proteobacteria, Bacteroidota and Actinobacteria in BSF larvae frass observed in this study was similar to previous findings (Gold et al. 2020; Wu et al. 2024; Wynants et al. 2019). Several biological functionalities can be generally attributed to these bacterial phyla: *e.g*. the degradation of recalcitrant molecules by Actinobacteria (Wang et al. 2016); consumption of easily degradable molecules by Firmicutes, as normally observed in the first stages of a composting process (Subirats et al. 2020) or accelerated fiber decomposition mediated by Bacteroidota (Zhong et al. 2020). However, without deepening the investigation in family level, genera or even into species that are active and viable in the frass—which can be performed by both bioinformatics, metagenomic and through microbial plating techniques—it is difficult to hypothesize the potential impacts of these frass types simply by observing the abundance of different bacterial groups. The same applies to the observed fungal groups, where a great abundance of Ascomycota and Basidiomycota was observed, groups that comprises thousands of species with varying functions within the soil‐plant continuum. Nevertheless, it is noteworthy how the diet with the highest olive pomace inclusion (Diet 84% OP) resulted in a frass with a generally lower diversity of both bacterial and fungal groups (Figures 2 and 4). Amplicon analysis is limited in its ability to accurately identify genera within samples; thus, even if such identification was available, understanding the specific functions of the observed microbial groups would remain limited without complementary genomic analysis. Therefore, future studies should combine these approaches with metagenomics, aiming to provide detailed information on the functionality of fungal and bacterial groups in frass fertilizers.

The inclusion of olive pomace in the diet of BSF larvae appeared to have led to a distinct microbial dynamic in the resulting frass fertilizers. This could be attributed to the potential phytotoxic properties of compounds present in olive pomace, which may select for more resistant groups of bacteria. When evaluating the composting of agricultural waste with olive mill wastewater, Abid and Sayadi (2006) reported that the presence of phenols in the wastewater had detrimental effects on the microbial activity, consequently compromising the composting process. Conversely, despite its toxic potential, Bouhia et al. (2022) reported > 90% reduction in the concentration of polyphenols after a co‐composting process of olive pomace with green waste, attributing this reduction especially to the shift in the microbial dynamics of the mixture, with fungi having great impacts in the transformation of the material and their components during the maturation stage. As reported by Lopes et al. (2024), frass maturation does not occur in a short bioconversion process such as the one carried out in this study (13 days), thus it is feasible to assume that the sole presence of olive pomace in the diets was responsible for the change in the microbial dynamics, as demonstrated in this study, but the potential effects in the transformation of substances such as phenolic compounds would l need a further decomposition step for be achieved.

The “bioenv” method followed by “ordistep” function that were applied to the full data set of both bacterial and fungal composition, selected the environmental variables that best explained the distribution of the microbial communities in the frass samples, reducing multicollinearity and eliminating redundant variables that did not add significant information to the model. As expected, the selected variables differed between bacteria and fungi, as each microbial group responds differently to varying environmental conditions and interacts in distinct ways with specific factors (Wang et al. 2024). The structure of the bacterial community was better differentiated in both the NMDS (Figure 2c) and the RDA (Figure 2d) compared to fungal groups (Figure 4c,d). However, of all variables analyzed, only Zn and Cu were considered highly relevant for such separation, explaining 34.8% of the variation observed in the distribution of bacteria. As demonstrated by Peng et al. (2022), Zn, Cu and other micronutrients interact with bacterial groups in the soil in complex ways, leading to shifts in community composition depending on their concentrations. According to Wang et al. (2024), the presence of micronutrients in the soil ‐ either naturally found or added through fertilizers—can explain the variations existing in the structure of microbial communities across different soils to a greater extent than geographic location, basic soil properties or macro‐elements. This can be attributed to several interacting factors, including the role of microelements as cofactors that support enzyme production in the soil‐plant continuum, as well as soil fertility traits such as pH, organic matter, or physical characteristics of the soil (Lopes et al. 2025). For instance, it was demonstrated by Dai et al. (2023) that single nutrients can contribute to the presence or absence of specific microbial groups in the soil, depending on their concentration.

When it comes to the fungal communities observed in the frass samples, the differentiation across samples was evident, albeit with less contrast compared to bacterial groups. In this case, four environmental variables were impactful (Mn, Ca, B and TOC), contributing to 47.1% of the variation observed in the community structure. The greater number of variables explaining fungal distribution than the bacterial distribution, may be due to the fact that bacteria tend to respond in a straightforward, linear manner to environmental factors such as the ones measured in this study (organic matter and both macro‐ and microelements) (Shepherd and Oliverio 2024). Therefore, only a few variables are typically sufficient to clearly distinguish and explain most of the observed variation in bacterial distribution. Fungi, on the other hand, appear to process information from multiple environmental gradients in a more complex manner. Thus, it is also possible that additional, not measured factors, influence the fungal community. If these variables would have been included, they may have improved our level of explanation of the variation observed in fungal distribution. It is noteworthy that isolating individual variables and examining their specific interaction with microbial communities is exceptionally complex, as both the soil and organic fertilizers such as frass ‐ and their interactions ‐ add complexity to the system. In this study, it was demonstrated that the concentration of micronutrients in olive pomace‐derived frass was similar to the values reported in the literature for frass derived from several other waste substrates (Lopes et al. 2022; Song et al. 2021). It is thus reasonable to assume that frass can contribute to shaping the soil communities due to its distinct micronutrient composition, which may, in turn, influence soil‐plant health (Noman et al. 2024). Therefore, despite its complexity, future studies should investigate the effects and interactions of prominent variables in frass with specific microbial communities, with special regard to groups considered as biostimulants (*e.g*. plant growth‐promoting rhizobacteria).

The presence of bacterial pathogens, targeting various organisms and functions, was significantly reduced in frass derived from the diet with the highest inclusion of olive pomace (Diet 84% OP) (Figure 3). The feed substrates used in this study were prepared on the same day, using the same waste streams from the same batch (vegetables and olive pomace), and the BSF larvae bioconversion took place concurrently. This suggests that the different inclusion rates of olive pomace were likely the only parameters affecting the abundance of these bacteria. The laboratory tests for the presence of *E. coli* and *Salmonella* spp. ‐ the two microorganisms specified in the European legislation for safe use of frass as a fertilizer (European Commission 2011) ‐ indicated the absence of both; however, both these bacteria as well as members of the *Clostridium* genus were detected in the frass microbiome through amplicon sequencing. This is likely a detection of DNA from inactive microorganisms spotted in the amplicon sequencing. Van Looveren et al. (2022) also heat‐treated frass at 70° C for 1 h and assessed the fate of *Salmonella* spp. (a mixture of *S. enterica* subsp. *enterica* serovar Infantis and *S. enterica* subsp. *enterica* serovar Typhimurium) and *Clostridium perfringens* in the fertilizer samples. Those authors reported a significant reduction in *Salmonella* spp, while only vegetative forms of *C. perfringens* were affected by the treatment and their endospores remained unaffected. Nevertheless, sequencing these microorganisms' RNA (which in turn do not account for inactive microbial DNA) in frass samples should be carried out in future studies, aiming to understand the real effect of BSF larvae on the survival and viability of these pathogenic microorganisms.

Similar observations were made regarding the presence of potentially pathogenic and non‐pathogenic fungal groups in frass samples, with a predominance of nectar/tap saprotrophs being observed, regardless of whether olive pomace had been included in the BSF larvae diet or not and the post‐treatment method adopted. frass from Diet 84% OP had the lowest pathogen richness of all diets (Figure 4a,b), suggesting again that the inclusion of olive pomace in the BSF larvae diet lead to selection of distinct groups of microorganisms, possible due to phytotoxic traits of this organic material (Schmidt et al. 2023). It is known that the bioconversion of contaminated waste streams by BSF larvae effectively inactivates several pathogenic microorganisms such as *Salmonella*, *E. coli* and even viruses like bacteriophages (Elhag et al. 2022; Erickson et al. 2004; Lopes et al. 2020; Lalander et al. 2025), while not affecting others (e.g. *Enterococcus* spp.), which might help explain the findings of this study for some groups of bacteria and fungi. In addition, it is noteworthy that all the pathogens analyzed through amplicon sequencing in the study were found in low concentrations in the frass samples, confirming that frass can be a hygienically safe fertilizer/soil amendment. Bohm et al.(2023) demonstrated the significant impact of BSF larvae in the bioconversion of biosolids contaminated with several pathogenic microorganisms (including *Salmonella* spp.) and antibiotic resistance genes (ARG) (e.g. *sul1*, *tetA*, *tetQ* and others), which are mostly inactivated or found in reduced concentrations after the process. However, not all pathogenic bacteria and targeted genes were impacted by the BSF larvae treatment in that study (corroborating the findings of this study as well), while some of the unaffected bacteria were likely harboring these ARG. This highlights the importance for future studies to address the viability of distinct bacterial and fungal groups in frass following BSF‐driven waste bioconversion, to clarify why some groups are affected while others remain unchanged.

## 5 Conclusions

The presence of olive pomace in the diets of black soldier fly larvae moderately reduced the bioconversion efficiency and larval yield, especially with a higher inclusion level of pomace (Diet 84% OP, olive pomace). However, larval proximate composition remained unchanged even at this inclusion level, with larvae presenting between 43 − 48% crude protein and 20%–23% ether extract. Some frass parameters changed, including reduced concentration of total organic carbon and increased concentration of phosphate with higher inclusion of pomace. Frass composition was unaffected by the posttreatment methods used (heat treatment or pelletization), demonstrating that these can be applied without changing the quality of the fertilizer. The microbial composition of the generated frass was significantly shaped by inclusion of olive pomace in the BSF larvae diet. Specifically, at the highest inclusion level of 84%, the abundance of potentially pathogenic fungi and bacteria in the resulting frass was significantly lower, suggesting that incorporating olive pomace into the BSF larvae diet improves frass sanitization during the bioconversion process, when compared to diets with lower olive pomace content. It was demonstrated in this study that a frass of high quality (in terms of plant nutrients) and hygienic safety (with respect to concentration of pathogenic microorganisms) can be produced through BSF larvae bioconversion of olive pomace.

## Author Contributions

**Ivã Guidini Lopes:** conceptualization (lead), investigation, methodology, formal analysis, data curation, writing (original draft), **Nathali Machado de Lima:** investigation, methodology, data curation, formal analysis, writing (original draft), **Teresa Ribeiro:** conceptualization, investigation, methodology, validation, writing (review and editing), **Daniel Murta:** conceptualization, resources, supervision, validation, writing (review and editing), **Jean Wan Hong Yong:** funding acquisition, resources, supervision, writing (review and editing), **Cecilia Lalander:** funding acquisition, resources, supervision, validation, writing (review and editing).

## Ethics Statement

The authors have nothing to report.

## Conflicts of Interest

The authors declare no conflicts of interest.

## Supporting information


**Figure S1:** Pelletizing machine used for pelletizing the fresh frass samples obtained from the bioconversion of three experimental diets containing or not olive pomace in their composition. Table S1. Composition of diets used to rear black soldier fly larvae in an industrial setting. **Table S2:** Macronutrient and micronutrient concentrations of black soldier fly larvae frass types derived from the bioconversion of distinct diets containing no (Diet 0% OP), low (35% OP) or high (84% OP) concentrations of olive pomace (OP). **Table S3:** Physico‐chemical characteristics and plant nutrients analyzed in black soldier fly larvae‐derived frass, obtained from three experimental diets containing 0% (Diet 0% OP), 35% (Diet 35% OP), or 84% (Diet 84% OP) olive pomace (OP), either fresh or submitted to two post‐treatments (heat treatment and pelletization). **Table S4:** Micronutrients analyzed in black soldier fly larvae‐derived frass, obtained from three experimental diets containing 0% (Diet 0% OP), 35% (Diet 35% OP), or 84% (Diet 84% OP) olive pomace (OP), either fresh or submitted to two post‐treatments (heat treatment and pelletization).

## Data Availability

The datasets generated and/or analyzed during the current study are not publicly available due to the fact that the study was conducted at Ingredient Odyssey SA ‐ Entogreen and a great part of it is considered sensible from an industrial perspective. However, analytical and microbiological data are available from the corresponding author on reasonable request.

## References

[mbo370180-bib-0001] Abbott, L. K. , L. M. Macdonald , M. T. F. Wong , M. J. Webb , S. N. Jenkins , and M. Farrell . 2018. “Potential Roles of Biological Amendments for Profitable Grain Production—A Review.” Agriculture, Ecosystems & Environment 256: 34–50. 10.1016/j.agee.2017.12.021.

[mbo370180-bib-0002] Abid, N. , and S. Sayadi . 2006. “Detrimental Effects of Olive Mill Wastewater on the Composting Process of Agricultural Wastes.” Waste Management 26: 1099–1107. 10.1016/j.wasman.2005.06.015. 16181778

[mbo370180-bib-0003] Ameixa, O. M. C. C. , M. Pinho , M. R. Domingues , and A. I. Lillebø . 2023. “Bioconversion of Olive Oil Pomace by Black Soldier Fly Increases Eco‐Efficiency in Solid Waste Stream Reduction Producing Tailored Value‐Added Insect Meals.” PLoS One 18: e0287986. 10.1371/journal.pone.0287986. 37478051 PMC10361471

[mbo370180-bib-0004] Antónia Nunes, M. , A. S. G. Costa , S. Bessada , et al. 2018. “Olive Pomace as a Valuable Source of Bioactive Compounds: A Study Regarding Its Lipid‐ and Water‐Soluble Components.” Science of the Total Environment 644: 229–236. 10.1016/j.scitotenv.2018.06.350. 29981971

[mbo370180-bib-0005] AOAC . 1920. Official Methods of Analysis of the Association of Official Analytical Chemists (1st ed.). AOAC International.

[mbo370180-bib-0006] AOAC . 1962. Official Methods of Analysis of the Association of Official Analytical Chemists (9th ed.). AOAC International.

[mbo370180-bib-0008] Azzaz, A. A. , M. Jeguirim , V. Kinigopoulou , et al. 2020. “Olive Mill Wastewater: From a Pollutant to Green Fuels, Agricultural and Water Source and Bio‐Fertilizer—Hydrothermal Carbonization.” Science of the Total Environment 733: 139314. 10.1016/j.scitotenv.2020.139314. 32446075

[mbo370180-bib-0009] Barragán‐Fonseca, K. Y. , L. O. Greenberg , G. Gort , M. Dicke , and J. J. A. van Loon . 2023. “Amending Soil With Insect Exuviae Improves Herbivore Tolerance, Pollinator Attraction and Seed Yield of *Brassica nigra* Plants.” Agriculture, Ecosystems & Environment 342: 108219. 10.1016/j.agee.2022.108219.

[mbo370180-bib-0010] Beesigamukama, D. , S. Subramanian , and C. M. Tanga . 2022. “Nutrient Quality and Maturity Status of Frass Fertilizer From Nine Edible Insects.” Scientific Reports 12: 7182. 10.1038/s41598-022-11336-z. 35505193 PMC9064968

[mbo370180-bib-0011] Bohm, K. , G. A. Hatley , B. H. Robinson , and M. J. Gutiérrez‐Ginés . 2023. “Analysis of Chemical and Phytotoxic Properties of Frass Derived From Black Soldier Fly‐Based Bioconversion of Biosolids.” Sustainability 15: 11526. 10.3390/su151511526.

[mbo370180-bib-0012] Bouhia, Y. , M. Hafidi , Y. Ouhdouch , et al. 2022. “Microbial Community Succession and Organic Pollutants Removal During Olive Mill Waste Sludge and Green Waste Co‐Composting.” Frontiers in Microbiology 12: 814553. 10.3389/fmicb.2021.814553. 35265049 PMC8899611

[mbo370180-bib-0013] Greg Caporaso , G. Ackermann , A. Apprill , et al. 2023. “EMP 16S Illumina Amplicon Protocol V.2.” Protocols.io. 10.17504/protocols.io.kqdg3dzzl25z/v2.

[mbo370180-bib-0014] Chen, Y. , M. Camps‐Arbestain , Q. Shen , B. Singh , and M. L. Cayuela . 2018. “The Long‐Term Role of Organic Amendments in Building Soil Nutrient Fertility: A Meta‐Analysis and Review.” Nutrient Cycling in Agroecosystems 111: 103–125. 10.1007/s10705-017-9903-5.

[mbo370180-bib-0015] Dai, Z. , X. Guo , J. Lin , et al. 2023. “Metallic Micronutrients Are Associated With the Structure and Function of the Soil Microbiome.” Nature Communications 14: 8456. 10.1038/s41467-023-44182-2. PMC10730613 38114499

[mbo370180-bib-0016] Difonzo, G. , M. Troilo , G. Squeo , A. Pasqualone , and F. Caponio . 2021. “Functional Compounds From Olive Pomace to Obtain High‐Added Value Foods—A Review.” Journal of the Science of Food and Agriculture 101: 15–26. 10.1002/jsfa.10478. 32388855

[mbo370180-bib-0017] Elhag, O. , Y. Zhang , X. Xiao , et al. 2022. “Inhibition of Zoonotic Pathogens Naturally Found in Pig Manure by Black Soldier Fly Larvae and Their Intestine Bacteria.” Insects 13: 66. 10.3390/insects13010066. 35055911 PMC8779730

[mbo370180-bib-0018] Erickson, M. C. , M. Islam , C. Sheppard , J. Liao , and M. P. Doyle . 2004. “Reduction of *Escherichia coli* O157:H7 and Salmonella Enterica Serovar Enteritidis in Chicken Manure by Larvae of the Black Soldier Fly.” Journal of Food Protection 67: 685–690. 10.4315/0362-028X-67.4.685. 15083719

[mbo370180-bib-0019] Esteves, C. , P. Fareleira , M. A. Castelo‐Branco , et al. 2022. “Black Soldier Fly Larvae Frass Increases the Soil's Residual Nutrient Content and Enzymatic Activity—A Lettuce Production Trial.” Journal of Insects as Food and Feed 8: 1431–1440. 10.3920/JIFF2022.0005.

[mbo370180-bib-0020] European Commission . 2011. “Commission Regulation (EU) No 142/2011 of 25 February 2011 Implementing Regulation (EC) No 1069/2009 of the European Parliament and of the Council Laying Down Health Rules as Regards Animal by‐Products and Derived Products Not Intended for Human Consumption and Implementing Council Directive 97/78/EC as Regards Certain Samples and Items Exempt From Veterinary Checks at the Border Under That Directive.” In Official Journal of the European Union L 54, 1–254.

[mbo370180-bib-0021] European Commission . 2017. “Commission Regulation (EU) 2017/1017 of 15 June 2017 Amending Annexes I, II, III, V and VI to Regulation (EC) No 2003/2003 of the European Parliament and of the Council Relating to Fertilisers.” In Official Journal of the European Union L (159, 1–92.

[mbo370180-bib-0022] European Commission . (2020). News 4 February (accessed on April 10, 2025). https://ec.europa.eu/info/news/producing-69-worlds-production-eu-largest-producer-olive-oil-2020-feb-04_en. 2020.

[mbo370180-bib-0023] European Commission . 2021. “Commission Regulation (EU) 2021/1925 of 5 November 2021 Amending Certain Annexes to Regulation (EU) No 142/2011 as Regards the Requirements for Placing on the Market of Certain Insect Products and the Adaptation of a Containment Method, L393.” In Official Journal of the European Union, 4–8. https://eur-lex.europa.eu/oj/direct-access.html.

[mbo370180-bib-0024] Gärttling, D. , and H. Schulz . 2022. “Compilation of Black Soldier Fly Frass Analyses.” Journal of Soil Science and Plant Nutrition 22: 937–943. 10.1007/s42729-021-00703-w.

[mbo370180-bib-0025] Gold, M. , F. von Allmen , C. Zurbrügg , J. Zhang , and A. Mathys . 2020. “Identification of Bacteria in Two Food Waste Black Soldier Fly Larvae Rearing Residues.” Frontiers in Microbiology 11: 2020. 33329446 10.3389/fmicb.2020.582867 PMC7719680

[mbo370180-bib-0026] Guidini Lopes, I. , V. Wiklicky , E. Ermolaev , and C. Lalander . 2023. “Dynamics of Black Soldier Fly Larvae Composting—Impact of Substrate Properties and Rearing Conditions on Process Efficiency.” Waste Management 172: 25–32. 10.1016/j.wasman.2023.08.045. 37708809

[mbo370180-bib-0027] ISO . 2002. ISO 6579:2002 Microbiology of Food and Animal Feeding Stuffs—Horizontal Method for the Detection of Salmonella spp. International Organization for Standardization.

[mbo370180-bib-0028] ISO . 2005. ISO 11866‐2:2005 Milk and Milk Products—Enumeration of Presumptive *Escherichia coli* ‐ Part 2: Most Probable Number Technique. International Organization for Standardization.

[mbo370180-bib-0029] Janssen, R. H. , J.‐P. Vincken , L. A. M. van den Broek , V. Fogliano , and C. M. M. Lakemond . 2017. “Nitrogen‐to‐Protein Conversion Factors for Three Edible Insects: *Tenebrio molitor*, *Alphitobius diaperinus*, and *Hermetia illucens* .” Journal of Agricultural and Food Chemistry 65: 2275–2278. 10.1021/acs.jafc.7b00471. 28252948 PMC5364430

[mbo370180-bib-0030] Khdair, A. , and G. Abu‐Rumman . 2020. “Sustainable Environmental Management and Valorization Options for Olive Mill Byproducts in the Middle East and North Africa (MENAa) Region.” Processes 8: 671. 10.3390/pr8060671.

[mbo370180-bib-0031] Lahti, L. , and S. Shetty (2012‐2019). “microbiome R package.”.

[mbo370180-bib-0032] Lindberg, L. , B. Vinnerås , and C. Lalander . 2025. “Process Efficiency and the Impact of Enzyme and Frass Additions in Black Soldier Fly Larvae Composting With Fibrous Plant‐Based Waste.” Journal of Environmental Chemical Engineering 13: 118061. 10.1016/j.jece.2025.118061.

[mbo370180-bib-0033] Van Looveren, N. , D. Vandeweyer , J. van Schelt , and L. Van Campenhout . 2022. “Occurrence of *Clostridium perfringens* Vegetative Cells and Spores Throughout an Industrial Production Process of Black Soldier Fly Larvae (*Hermetia illucens*).” Journal of Insects as Food and Feed 8: 399–407. 10.3920/JIFF2021.0073.

[mbo370180-bib-0034] Lopes, I. G. , M. Gómez‐Brandón , N. Praeg , et al. 2025. “Bugbook: Critical Considerations for Evaluating and Applying Insect Frass.” Journal of Insects as Food and Feed 11, no. 18: 507–534. 10.1163/23524588-bja10254.

[mbo370180-bib-0035] Lopes, I. G. , C. Lalander , R. M. Vidotti , and B. Vinnerås . 2020. “Reduction of Bacteria in Relation to Feeding Regimes When Treating Aquaculture Waste in Fly Larvae Composting.” Frontiers in Microbiology 11: 1616. 10.3389/fmicb.2020.01616. 32765458 PMC7378744

[mbo370180-bib-0036] Lopes, I. G. , V. Wiklicky , B. Vinnerås , J. W. H. Yong , and C. Lalander . 2024. “Recirculating Frass From Food Waste Bioconversion Using Black Soldier Fly Larvae: Impacts on Process Efficiency and Product Quality.” Journal of Environmental Management 366: 121869. 10.1016/j.jenvman.2024.121869. 39029172

[mbo370180-bib-0037] Lopes, I. G. , J. W. Yong , and C. Lalander . 2022. “Frass Derived From Black Soldier Fly Larvae Treatment of Biodegradable Wastes: A Critical Review and Future Perspectives.” Waste Management 142: 65–76. 10.1016/j.wasman.2022.02.007. 35176600

[mbo370180-bib-0038] López‐Mosquera, M. E. , F. Cabaleiro , M. J. Sainz , A. López‐Fabal , and E. Carral . 2008. “Fertilizing Value of Broiler Litter: Effects of Drying and Pelletizing.” Bioresource Technology 99: 5626–5633. 10.1016/j.biortech.2007.10.034. 18609765

[mbo370180-bib-0039] Martin, M. 2011. “Cutadapt Removes Adapter Sequences From High‐Throughput Sequencing Reads.” EMBnet.journal 17: 10. 10.14806/ej.17.1.200.

[mbo370180-bib-0040] Mostafaie, A. , A. R. R. Silva , J. N. Pinto , et al. 2025. “Towards Circularity for Agro‐Waste: Minimal Soil Hazards of Olive Pomace Bioconverted Frass by Insect Larvae as an Organic Fertilizer.” Journal of Environmental Management 375: 124151. 10.1016/j.jenvman.2025.124151. 39874695

[mbo370180-bib-0041] Muscolo, A. , T. Papalia , G. Settineri , F. Romeo , and C. Mallamaci . 2019. “Three Different Methods for Turning Olive Pomace in Resource: Benefits of the End Products for Agricultural Purpose.” Science of the Total Environment 662: 1–7. 10.1016/j.scitotenv.2019.01.210. 30682711

[mbo370180-bib-0042] Nilsson, R. H. , K.‐H. Larsson , A. F. S. Taylor , et al. 2019. “The Unite Database for Molecular Identification of Fungi: Handling Dark Taxa and Parallel Taxonomic Classifications.” Nucleic Acids Research 47: D259–D264. 10.1093/nar/gky1022. 30371820 PMC6324048

[mbo370180-bib-0043] Noman, M. , T. Ahmed , J. Wang , and J. C. White . 2024. “Micronutrient–Microbiome Interplay: A Critical Regulator of Soil‐Plant Health.” Trends in Microbiology 32: 319–320. 10.1016/j.tim.2024.02.008. 38395702

[mbo370180-bib-0044] Oksanen, J. , F. G. Blanchet , M. Friendly , R. Kindt , P. Legendre , and D. McGlinn , 2020. Vegan: Community Ecology Package, 2.5–7. Available from: https://CRAN.R-project.org/package=vegan.

[mbo370180-bib-0045] Osuch, B. , M. Barszcz , and D. Tomaszewska‐Zaremba . 2024. “The Potential of Black Soldier Fly (≪I≫*Hermetia illucens*≪/I≫ L.) Larvae in Chicken and Swine Nutrition: A Review.” Journal of Animal and Feed Sciences 33: 454–468. 10.22358/jafs/192511/2024.

[mbo370180-bib-0046] Peng, Z. , C. Liang , M. Gao , et al. 2022. “The Neglected Role of Micronutrients in Predicting Soil Microbial Structure.” NPJ Biofilms and Microbiomes 8: 103. 10.1038/s41522-022-00363-3. 36575178 PMC9794713

[mbo370180-bib-0047] Põlme, S. , K. Abarenkov , R. Henrik Nilsson , et al. 2020. “Fungaltraits: A User‐Friendly Traits Database of Fungi and Fungus‐Like Stramenopiles.” Fungal Diversity 105: 1–16. 10.1007/s13225-020-00466-2.

[mbo370180-bib-0048] Praeg, N. , and T. Klammsteiner . 2024. “Primary Study on Frass Fertilizers From Mass‐Reared Insects: Species Variation, Heat Treatment Effects, and Implications for Soil Application at Laboratory Scale.” Journal of Environmental Management 356: 120622. 10.1016/j.jenvman.2024.120622. 38513580

[mbo370180-bib-0049] Quast, C. , E. Pruesse , P. Yilmaz , et al. 2012. “The SILVA Ribosomal RNA Gene Database Project: Improved Data Processing and Web‐Based Tools.” Nucleic Acids Research 41: D590–D596. 10.1093/nar/gks1219. 23193283 PMC3531112

[mbo370180-bib-0050] R. Core Team . 2023. R: A Language and Environment for Statistical Computing. R Foundation for Statistical Computing. https://www.R-project.org/.

[mbo370180-bib-0051] Ramzy, R. R. , M. A. El‐Dakar , D. Wang , and H. Ji . 2022. “Conversion Efficiency of Lignin‐Rich Olive Pomace to Produce Nutrient‐Rich Insect Biomass by Black Soldier Fly Larvae, *Hermetia illucens* .” Waste and Biomass Valorization 13: 893–903. 10.1007/s12649-021-01546-3.

[mbo370180-bib-0052] Sarlaki, E. , A. M. Kermani , M. H. Kianmehr , et al. 2021. “Improving Sustainability and Mitigating Environmental Impacts of Agro‐Biowaste Compost Fertilizer by Pelletizing‐Drying.” Environmental Pollution 285: 117412. 10.1016/j.envpol.2021.117412. 34051566

[mbo370180-bib-0053] Schmidt, L. , O. D. Prestes , P. R. Augusti , and J. C. Fonseca Moreira . 2023. “Phenolic Compounds and Contaminants in Olive Oil and Pomace—A Narrative Review of Their Biological and Toxic Effects.” Food Bioscience 53: 102626. 10.1016/j.fbio.2023.102626.

[mbo370180-bib-0054] Shepherd, R. M. , and A. M. Oliverio . 2024. “Micronutrients Modulate the Structure and Function of Soil Bacterial Communities.” Soil Biology and Biochemistry 192: 109384. 10.1016/j.soilbio.2024.109384.

[mbo370180-bib-0055] Song, S. , A. W. L. Ee , J. K. N. Tan , et al. 2021. “Upcycling Food Waste Using Black Soldier Fly Larvae: Effects of Further Composting on Frass Quality, Fertilising Effect and Its Global Warming Potential.” Journal of Cleaner Production 288: 125664. 10.1016/j.jclepro.2020.125664.

[mbo370180-bib-0056] Subirats, J. , R. Murray , A. Scott , C. H.‐F. Lau , and E. Topp . 2020. “Composting of Chicken Litter From Commercial Broiler Farms Reduces the Abundance of Viable Enteric Bacteria, Firmicutes, and Selected Antibiotic Resistance Genes.” Science of the Total Environment 746: 141113. 10.1016/j.scitotenv.2020.141113. 32768779

[mbo370180-bib-0057] Tapia‐Quirós, P. , M. F. Montenegro‐Landívar , M. Reig , et al. 2020. “Olive Mill and Winery Wastes as Viable Sources of Bioactive Compounds: A Study on Polyphenols Recovery.” Antioxidants 9: 1074. 10.3390/antiox9111074. 33139671 PMC7694004

[mbo370180-bib-0058] Wang, C. , D. Dong , H. Wang , et al. 2016. “Metagenomic Analysis of Microbial Consortia Enriched From Compost: New Insights Into the Role of Actinobacteria in Lignocellulose Decomposition.” Biotechnology for Biofuels 9: 22. 10.1186/s13068-016-0440-2. 26834834 PMC4731972

[mbo370180-bib-0059] Wang, X. , L. Wang , B. Wu , et al. 2024. “Neglected Role of Microelements in Determining Soil Microbial Communities and Fruit Micronutrients in Loquat Orchards.” Frontiers in Microbiology 15: 2024. 10.3389/fmicb.2024.1447921 PMC11373571 39234550

[mbo370180-bib-0060] Wilkinson, L. 2011. “ggplot2: Elegant Graphics for Data Analysis by WICKHAM, H.” Biometrics 67: 678–679. 10.1111/j.1541-0420.2011.01616.x.

[mbo370180-bib-0061] Wu, K. , J. Zhang , Q. Zhang , et al. 2015. “Plant Phenolics Are Detoxified by Prophenoloxidase in the Insect Gut.” Scientific Reports 5: 16823. 10.1038/srep16823. 26592948 PMC4655367

[mbo370180-bib-0062] Wu, N. , Y. Ma , X. Yu , et al. 2024. “Black Soldier Fly Larvae Bioconversion and Subsequent Composting Promote Larval Frass Quality During Pig and Chicken Manure Transformation Process.” Bioresource Technology 402: 130777. 10.1016/j.biortech.2024.130777. 38701978

[mbo370180-bib-0063] Wynants, E. , L. Frooninckx , S. Crauwels , et al. 2019. “Assessing the Microbiota of Black Soldier Fly Larvae (*Hermetia illucens*) Reared on Organic Waste Streams on Four Different Locations at Laboratory and Large Scale.” Microbial Ecology 77: 913–930. 10.1007/s00248-018-1286-x. 30430196

[mbo370180-bib-0064] Zhong, X.‐Z. , X.‐X. Li , Y. Zeng , S.‐P. Wang , Z.‐Y. Sun , and Y.‐Q. Tang . 2020. “Dynamic Change of Bacterial Community During Dairy Manure Composting Process Revealed by High‐Throughput Sequencing and Advanced Bioinformatics Tools.” Bioresource Technology 306: 123091. 10.1016/j.biortech.2020.123091. 32169511

