# Supplementary material for: Olive Pomace Inclusion Alters the Microbial Community of Black Soldier Fly Larvae Frass While Maintaining Fertilizer Quality
Source: Microbiologyopen. 2025 Nov 30;14(6):e70180. doi: 10.1002/mbo3.70180 (PMC12665153; doi:10.1002/mbo3.70180)
Supplement: Supplementary file 1 — Figure S1: Pelletizing machine used for pelletizing the fresh frass samples obtained from the bioconversion of three experimental diets containing or not olive pomace in their composition. Table S1. Composition of diets used to rear black soldier fly larvae in an industrial setting. Table S2: Macronutrient and micronutrient concentrations of black soldier fly larvae frass types derived from the bioconversion of distinct diets containing no (Diet 0% OP), low (35% OP) or high (84% OP) concentrations of olive pomace (OP). Table S3: Physico‐chemical characteristics and plant nutrients analyzed in black soldier fly larvae‐derived frass, obtained from three experimental diets containing 0% (Diet 0% OP), 35% (Diet 35% OP), or 84% (Diet 84% OP) olive pomace (OP), either fresh or submitted to two post‐treatments (heat treatment and pelletization). Table S4: Micronutrients analyzed in black soldier fly larvae‐derived frass, obtained from three experimental diets containing 0% (Diet 0% OP), 35% (Diet 35% OP), or 84% (Diet 84% OP) olive pomace (OP), either fresh or submitted to two post‐treatments (heat treatment and pelletization). [file MBO3-14-e70180-s001.docx]

**Supplementary Data**

**Olive pomace inclusion alters the microbial community of black soldier fly larvae frass while maintaining fertilizer quality**

Ivã Guidini Lopes, Nathali Machado de Lima, Teresa Ribeiro, Daniel Murta, Jean Wan Hong Yong, Cecilia Lalander

This supplementary data contains information on the physico-chemical composition of the three experimental diets used in the study for rearing black soldier fly larvae (BSFL), with or without olive pomace (Table S1). In addition, complementary information on the plant nutrient composition of the frass fertilizers obtained with the bioconversion of the three experimental diets is presented in Table S2. Tables S3 and S4 are showing the physico-chemical characteristics of the frass fertilizers obtained with the study, regarding the real values of each variable, in a non-factorial way. Finally, Figure S1 is a picture of the pelletizing machine used for the pelleting of the frass fertilizers.

**Table S1**. Composition of diets used to rear black soldier fly larvae in an industrial setting. Diet 0% OP was composed solely by plant-based by-products (vegetable cuttings) and no olive pomace, while Diet 35% OP and Diet 84% OP had olive pomace (OP) as part of its composition.

|  | Diet 0% OP | Diet 35% OP | Diet 84% OP |
| --- | --- | --- | --- |
| *Proximate composition* |  |  |  |
| Dry matter (%) | 30.3 | 32.5 | 34.4 |
| Crude protein (%) | 6.18 | 5.58 | 7.09 |
| Crude fat (%) | 1.8 | 2.41 | 3.5 |
| Crude Fiber (%) | 9.6 | 12.1 | 13.6 |
| Ash (%) | 1.9 | 2.41 | 3.12 |
|  |  |  |  |
| *By-products used* |  |  |  |
| Olive pomace (%) | - | 35.5 | 83.6 |
| Red peppers (%) | 31.0 | 0.5 | 1.4 |
| Pumpkin (%) | 30.8 | 0.5 | 1.4 |
| Broccoli (%) | 7.4 | 0.2 | 0.6 |
| Zucchini (%) | 4.9 | 2.6 | 0.3 |
| Grains (%) | 25.3 | 21.3 | 12.4 |
| Water (%) | 0.6 | 39.4 | 0.3 |

**Table S2.** Macronutrient and micronutrient concentrations of black soldier fly larvae frass types derived from the bioconversion of distinct diets containing no (Diet 0% OP), low (35% OP) or high (84% OP) concentrations of olive pomace (OP).

|  | CaO  (%_DM_) | MgO  (%_DM_) | Cu  (mg kg^-1^ _DM_) | Mn  (mg kg^-1^ _DM_) | Zn  (mg kg^-1^ _DM_) | Na  (mg kg^-1^ _DM_) |
| --- | --- | --- | --- | --- | --- | --- |
| *Diet* |  |  |  |  |  |  |
| 0% OP | 0.62^b^ | 0.64^b^ | 39.2^a^ | 56.9^b^ | 49.0^b^ | 0.06 |
| 35% OP | 0.82^ab^ | 0.95^ab^ | 34.5^b^ | 72.8^ab^ | 52.2^b^ | 0.10 |
| 84% OP | 1.12^a^ | 1.28^a^ | 31.5^c^ | 102.4^a^ | 72.3^a^ | 0.14 |
| *Type* |  |  |  |  |  |  |
| Fresh | 0.86 | 0.99 | 35.5 | 78.4 | 60.6 | 0.10 |
| Heat | 0.91 | 1.01 | 32.9 | 83.1 | 63.2 | 0.11 |
| Pellet | 0.78 | 0.87 | 36.9 | 70.6 | 49.6 | 0.09 |
|  |  |  |  |  |  |  |
| *Statistics* |  |  |  |  |  |  |
| Diet | 211.8^***^ | 57.6^**^ | 65.4^*^ | 232.8^**^ | 148.3^**^ | 87.5^ns^ |
| Type | 14.4^ns^ | 2.8^ns^ | 18.3^ns^ | 17.5^ns^ | 47.8^ns^ | 16.7^ns^ |
| Interaction | 11.3^ns^ | 11.9^ns^ | 27.9^ns^ | 21.8^ns^ | 34.7^ns^ | 2.3^ns^ |

**CaO**: calcium oxide; **MgO**: magnesium oxide; **Cu**: copper; **Mn**: manganese; **Zn**: zinc; **Na**: sodium. Distinct superscript letters indicate significant differences according to the Tukey’s test at a 5% probability level. *****p < 0.05; ******p < 0.01; *******p < 0.001; **ns**: not significant.

**Table S3.** Physico-chemical characteristics and plant nutrients analyzed in black soldier fly larvae-derived frass, obtained from three experimental diets containing 0% (Diet 0% OP), 35% (Diet 35% OP), or 84% (Diet 84% OP) olive pomace (OP), either fresh or submitted to two post-treatments (heat treatment and pelletization). Values are presented as mean ± standard deviation.

| Diet | Type | DM | OM | TOC | N_T_ | P_2_O_5_ | K_2_O | C/N | pH | EC |
| --- | --- | --- | --- | --- | --- | --- | --- | --- | --- | --- |
|  |  | *%* | *----------------------------- %_DM_ ----------------------------* | | | | |  |  | dS/m |
| *0% OP* | Fresh | 76.3 ± 1.1 | 84.0 ± 0.2 | 43.3 ± 0.2 | 3.6 ± 0.0 | 1.6 ± 0.1 | 6.1 ± 0.1 | 12.1 ± 0.1 | 8.8 ± 0.1 | 10.5 ± 0.3 |
|  | Pelletized | 65.7 ± 0.6 | 83.1 ± 0.3 | 43.4 ± 0.3 | 3.7 ± 0.1 | 1.6 ± 0.0 | 6.0 ± 0.1 | 11.8 ± 0.3 | 8.8 ± 0.3 | 10.7 ± 0.1 |
|  | Heat-treated | 64.7 ± 1.5 | 83.7 ± 0.6 | 41.2 ± 0.4 | 3.6 ± 0.2 | 2.3 ± 0.2 | 5.7 ± 0.2 | 11.6 ± 0.5 | 9.2 ± 0.0 | 9.7 ± 0.1 |
| *35% OP* | Fresh | 47.7 ± 2.1 | 81.7 ± 0.2 | 37.7 ± 0.1 | 3.6 ± 0.1 | 4.1 ± 0.2 | 6.1 ± 0.2 | 10.6 ± 0.4 | 8.9 ± 0.1 | 9.6 ± 0.3 |
|  | Pelletized | 65.8 ± 0.4 | 83.6 ± 0.2 | 43.2 ± 0.3 | 3.7 ± 0.1 | 1.4 ± 0.0 | 5.9 ± 0.2 | 11.7 ± 0.5 | 8.4 ± 0.3 | 11.2 ± 0.1 |
|  | Heat-treated | 65.4 ± 1.6 | 83.4 ± 0.4 | 41.2 ± 0.3 | 3.3 ± 0.1 | 2.0 ± 0.2 | 5.4 ± 0.2 | 12.5 ± 0.1 | 9.0 ± 0.2 | 9.8 ± 0.5 |
| *84% OP* | Fresh | 75.7 ± 2.8 | 84.6 ± 0.1 | 41.7 ± 0.3 | 3.2 ± 0.0 | 2.3 ± 0.1 | 5.7 ± 0.1 | 13.2 ± 0.1 | 9.0 ± 0.1 | 10.3 ± 0.3 |
|  | Pelletized | 43.8 ± 1.8 | 81.3 ± 0.2 | 37.7 ± 0.1 | 3.7 ± 0.1 | 4.4 ± 0.1 | 6.5 ± 0.1 | 10.2 ± 0.3 | 8.8 ± 0.1 | 9.7 ± 0.3 |
|  | Heat-treated | 65.8 ± 5.1 | 83.0 ± 0.2 | 39.1 ± 0.1 | 3.3 ± 0.1 | 3.9 ± 0.1 | 5.8 ± 0.1 | 11.8 ± 0.2 | 8.8 ± 0.1 | 11.2 ± 0.5 |

**DM**: dry matter; **OM**: organic matter; **TOC**: total organic carbon; **N_T_**: total nitrogen; **P_2_O_5_**: phosphate; **K_2_O**: potassium oxide; **C/N**: carbon to nitrogen ratio; **EC**: electrical conductivity.

**Table S4.** Micronutrients analyzed in black soldier fly larvae-derived frass, obtained from three experimental diets containing 0% (Diet 0% OP), 35% (Diet 35% OP), or 84% (Diet 84% OP) olive pomace (OP), either fresh or submitted to two post-treatments (heat treatment and pelletization). Values are presented as mean ± standard deviation.

| Diet | Type | CaO | MgO | Cu | Mn | Zn | NaO |
| --- | --- | --- | --- | --- | --- | --- | --- |
|  |  | *-----(%_DM_) -----* | | *--------------- (mg/kg_DM_) ---------------* | | | |
| *0% OP* | Fresh | 0.5 ± 0.0 | 0.7 ± 0.0 | 40.4 ± 1.5 | 48.0 ± 2.2 | 52.4 ± 2.9 | 0.05 ± 0.0 |
|  | Pelletized | 0.5 ± 0.0 | 0.5 ± 0.3 | 40.5 ± 1.4 | 46.7 ± 1.3 | 42.6 ± 0.1 | 0.05 ± 0.0 |
|  | Heat-treated | 0.8 ± 0.1 | 0.8 ± 0.1 | 36.8 ± 1.9 | 76.0 ± 4.1 | 51.9 ± 0.5 | 0.09 ± 0.0 |
| *35% OP* | Fresh | 1.2 ± 0.1 | 1.5 ± 0.1 | 29.5 ± 0.7 | 110.0 ± 0.0 | 66.0 ± 1.9 | 0.17 ± 0.0 |
|  | Pelletized | 0.5 ± 0.0 | 0.6 ± 0.0 | 39.6 ± 1.3 | 41.7 ± 1.2 | 41.0 ± 0.8 | 0.05 ± 0.0 |
|  | Heat-treated | 0.8 ± 0.0 | 0.8 ± 0.0 | 34.5 ± 1.0 | 66.7 ± 3.4 | 49.5 ± 1.1 | 0.08 ± 0.0 |
| *84% OP* | Fresh | 0.9 ± 0.1 | 0.8 ± 0.0 | 36.5 ± 0.7 | 77.3 ± 6.6 | 63.6 ± 2.5 | 0.09 ± 0.0 |
|  | Pelletized | 1.3 ± 0.0 | 1.6 ± 0.0 | 30.7 ± 1.0 | 123.3 ± 4.7 | 65.3 ± 2.1 | 0.18 ± 0.0 |
|  | Heat-treated | 1.2 ± 0.1 | 1.4 ± 0.1 | 27.3 ± 0.3 | 106.7 ± 4.7 | 88.2 ± 5.8 | 0.16 ± 0.0 |

**CaO**: calcium oxide; **MgO**: magnesium oxide; **Cu**: copper; **Mn**: manganese; **Zn**: zinc; **NaO**: sodium.


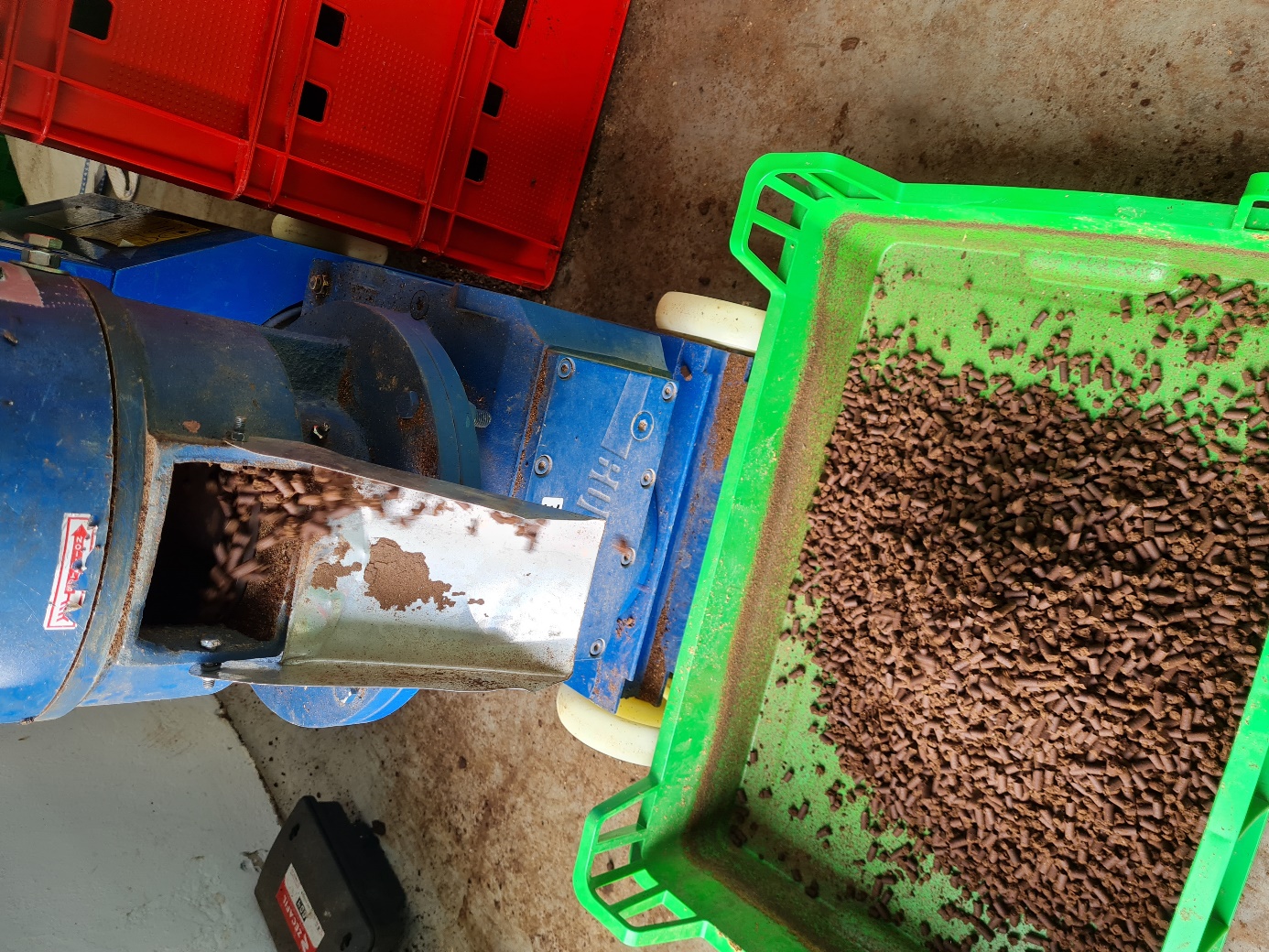


**Figure S1.** Pelletizing machine used for pelletizing the fresh frass samples obtained from the bioconversion of three experimental diets containing or not olive pomace in their composition. Frass was poured into the pelletizing machine slowly and continuously in order to ensure the best homogenization possible in relation to pellet size and production.
